# Glioblastoma progression is assisted by induction of immunosuppressive function of pericytes through interaction with tumor cells

**DOI:** 10.18632/oncotarget.19804

**Published:** 2017-08-02

**Authors:** Rut Valdor, David García-Bernal, Carlos Bueno, Mónica Ródenas, José M. Moraleda, Fernando Macian, Salvador Martínez

**Affiliations:** ^1^ Internal Medicine Department, Medicine School, University of Murcia, Murcia, Spain; ^2^ Brain Regionalization and Development Gene Unit, Biomedical Research Institute of Murcia (IMIB-Arrixaca), Murcia, Spain; ^3^ Hematopoietic Transplant and Cellular Therapy Unit, Hematology Service, Virgen de la Arrixaca Clinical University Hospital, Biomedical Research Institute of Murcia (IMIB-Arrixaca), Murcia, Spain; ^4^ Department of Pathology, Institute for Aging Studies, Albert Einstein College of Medicine, Bronx, New York, USA; ^5^ Instituto de Neurociencias CSIC-UMH and Human Anatomy Department, Instituto de Neurociencias CSIC-UMH, School of Medicine, University Miguel Hernandez, CIBERSAM of ISCIII, Alicante, Spain

**Keywords:** glioblastoma multiforme, brain perivascular cells, tumor, immunotolerance, T cells

## Abstract

The establishment of immune tolerance during Glioblastoma Multiforme (GBM) progression, is characterized by high levels expression of anti-inflammatory cytokines, which suppress the function of tumor assocciated myeloid cells, and the activation and expansion of tumor antigen specific T cells. However, the mechanisms underlying the failed anti-tumor immune response around the blood vessels during GBM, are poorly understood. The consequences of possible interactions between cancer cells and the perivascular compartment might affect the tumor growth. In this work we show for the first time that GBM cells induce immunomodulatory changes in pericytes in a cell interaction-dependent manner, acquiring an immunosuppresive function that possibly assists the evasion of the anti-tumor immune response and consequently participates in tumor growth promotion. Expression of high levels of anti-inflammatory cytokines was detected *in vitro* and *in vivo* in brain pericytes that interacted with GBM cells (GBC-PC). Furthermore, reduction of surface expression of co-stimulatory molecules and major histocompatibility complex molecules in GBC-PC correlated with a failure of antigen presentation to T cells and the acquisition of the ability to supress T cell responses. *In vivo*, orthotopic xenotransplant of human glioblastoma in an immunocompetent mouse model showed significant GBM cell proliferation and tumor growth after the establishment of interspecific immunotolerance that followed GMB interaction with pericytes.

## INTRODUCTION

Glioblastoma multiforme (GBM) is a highly invasive cancer that is characterized by changes in cerebral vessels and the gradual invasion of surrounding tissues along the perivascular space [1, 2]. In the last years, several immune regulators have been shown to contribute to GBM progression as immunossupressive mechanisms, including recruitment of tumor-associated macrophages (TAMs) and changes of the expression patterns of cytokines that modulate the anti-tumor T cell responses [3–6]. However, the origin of these initial mechanisms underlying the suppression of anti-tumor immune responses around the blood vessel, where GBM initiates and progresses, are poorly understood. Previous works suggest that glioblastoma cells can differentiate into cells of tumor vessels, providing a means of tumor vascularization [7]; and that the tumorogenic phenotype can be transferred from the tumor cell to host cells around the blood vessels during GBM progression [8, 9]. Thus, to better understand these processes, it is essential to determine the consequences of possible interactions between cancer cells and cells in the perivascular compartments.

Small blood vessels are composed of two different but interdependent cellular compartments, endothelial cells (ECs) and pericytes [10, 11]. Pericytes are perivascular stromal cells that are located on the abluminal vessel wall of brain capillaries and regulate vascular tone and morphology, in a similar way as vascular smooth muscle cells do in big blood vessels [10, 12]. Pericytes may have stem cell properties and represent an immunological defense in the brain [13, 14]. Indeed, they have phagocytic activity and express numerous macrophage markers, supporting an ability to acquire functional properties of macrophages [9, 15].

Immune activation of brain pericytes, which act as mediators of neuroinflammation, specifically leads to the expression of several pro-inflammatory cytokines, co-stimulatory molecules and Major Histocompatibility Complex (MHC) molecules, which may modulate T cell responses [16–19]. The role of pericytes in the regulation of T cell function is poorly understood. Some authors described the ability of pericytes pre-activated by inflammatory challenge to present antigens on MHC to T cells, modulating the responses of different T cell populations, including suppressive regulatory T cells [16, 20, 21]. However, it is not well known if brain pericytes are immunologically protective in response to tumor formation or if, by contrast, they might fail to activate antigenic T cell responses, preventing tumor clearance.

The lack of pericytes promotes endothelial hyperplasia and abnormal vascular morphogenesis, which contributes to increased transendothelial permeability [9, 22], Pericytes can also regulate the expression of cytokines, chemokines and proteases in the tumor cell niche, which may promote immunosuppression, tumor angiogenesis, growth and metastasis [23, 24]. Therefore, pericytes might provide a critical element for the local control of both vascular-tumor cell interaction and the modulation of the anti-tumor immune response. In this work, we show that glioblastoma cells induce immunotolerant properties in brain pericytes. This may explain, at least in part, why GBM cells are not detected by the immune system during perivascular inflitration and tumor growth. Thus interference with the immunossuppressive function of pericytes may represent a novel target for the development of new therapies against GBM.

With our work, we try to address the mechanisms that might explain how the interaction of glioblastoma cells with pericytes allows tumor to grow and infiltrate brain parenchyma; and to specifically determine the relevance of this intercellular interaction in the modulation of the anti-glioblastoma immune response. A non-immunosuppressed mouse Glioblastoma model has been defined as a useful tool to study human Glioblastoma [25]. The use of xenografts of human glioblastoma cells makes our experimental model more relevant to demonstrate an immunoregulatory mechanism activated by human glioblastoma. We show that, even under those conditions, immunocompetent C57BL/6 wild type mice cannot reject human glioblastoma tumor, likely due to the potent ability of glioblastoma cells to induce immune tolerance. Our purpose was to use this system to identify those mechanisms that allow glioblastoma cells to induce such a profound tolerant state, and may also represent a new approach to develop new experimental designs to study GBM-immune system interactions.

## RESULTS

### Pericytes interacting with GBM cells show an anti-inflammatory phenotype

**Figure 1:**
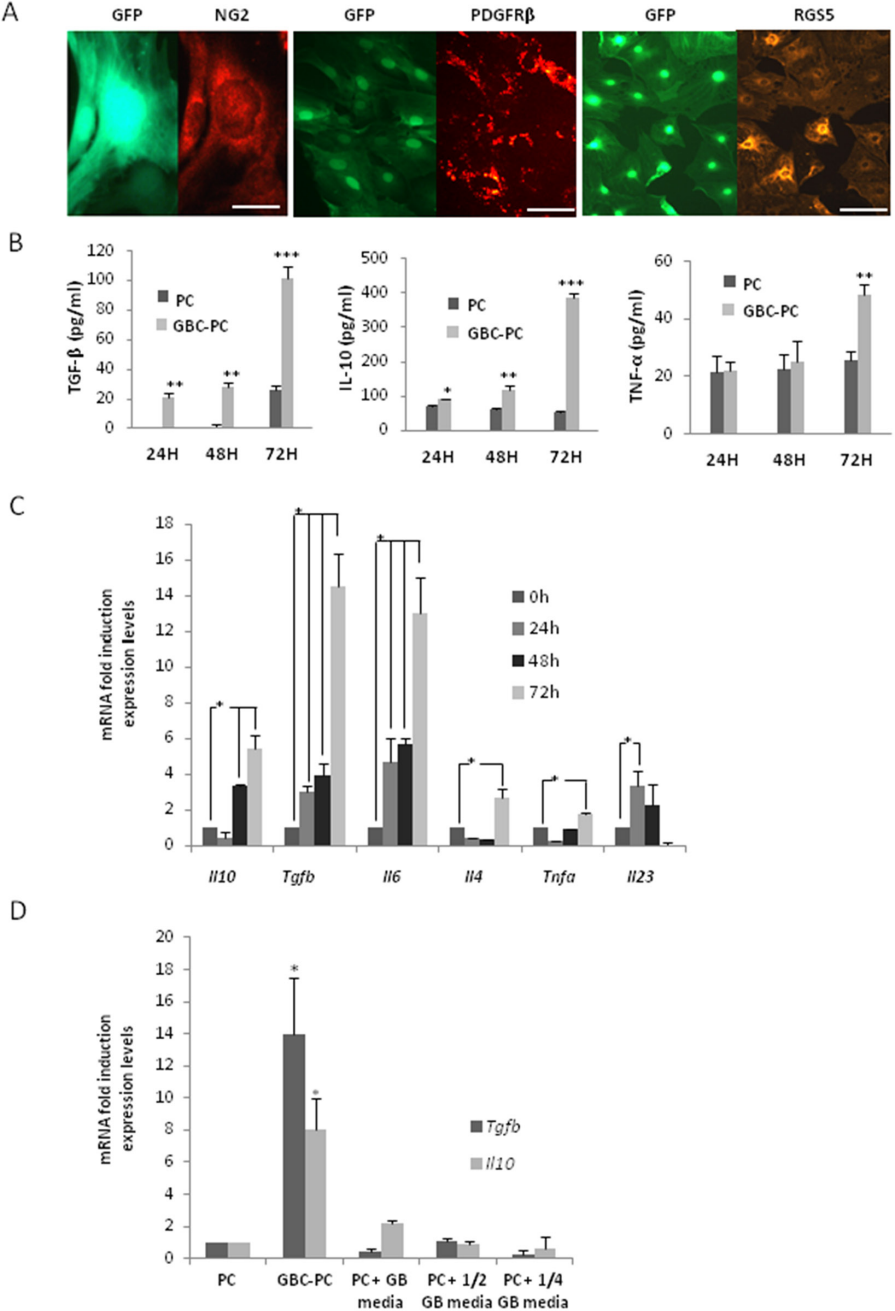
Pericytes interacting with GBM cells show an anti-inflammatory phenotype **(A)** Expression of pericyte markers in pericytes expressing GFP. NG2 (scale bar, 50 μm), PDGFRβ and RGS5 (scale bar, 100 μm). The images are representative of at least, three independent experiments. **(B)** ELISA measuring IL-10, TGF-β and TNF-α levels in pericytes co-cultured with Glioblastoma cells (GBC-PC) at different time points, and at basal levels in control pericytes (PC), ** p<0.01 or ***p<0.001. All data represent mean ± Standard Deviation obtained from at least three independent experiments. **(C)** Quantitative analysis of cytokine mRNA expression in GBC-PC at different time points. Results are presented relative to those of basal levels in control pericytes at each time point, and normalized to the housekeeping reference gene expression, * p<0.01. All data represents mean ± Standard Deviation obtained from at least four independent experiments. **(D)** Quantitative analysis of IL-10 and TGF-β mRNA expression in pericytes (PC), after 72 hours in different conditions of culture (pericytes in presence of GBM cells: GBC-PC; pericytes in presence of several dilutions of GBM conditioned media: PC + ½, ½ GB media. Results are presented relative to those of basal levels in control pericytes at 72 hours of culture, and normalized to the housekeeping reference gene expression, * p<0.01. All data represents mean ± Standard Deviation obtained from at least, five independent experiments using U373 and U87 GBM lines independently.

The immune function of pericytes in response to tumor cells remains unknown. To characterize if brain pericytes may acquire an anti-inflammatory function in response to their interaction with GBM cells (glioblastoma-conditioned-pericytes: GBC-PC) during tumor progression, we determined first if changes in the expression levels of cytokines would occur in response to the interaction of pericytes with GBM cells *in vitro*. Purification of brain pericytes from transgenic mice that express actin fusioned to GFP (C57Bl/6-Tg(ACTB-EGFP)1Osb/J), was confirmed by immunofluorescence after five passages [9, 26, 27], using well characterized pericyte markers [28, 29], such as NG2, PDGFR-β, and RGS-5 (Figure 1A). Staining for control markers of astrocytes, microglia and endothelial cells was negative (not shown), which ensures that none other cell type without stem cells properties survived in a culture without specific growth factors [26, 27]. Analyses of cytokines secreted from pericytes co-cultured with a GBM human cell line (GBC-PC), revealed a significant increase in the production of IL-10 and TGF-β after 72 hours compared to basal levels of control pericytes (Figure 1B). The expression of pro-inflammatory cytokines, such as IL-1, IL-23 and IL-12 was, however, hardly detectable (not shown). GBC-PC also produced the pro-inflammatory cytokine TNF-α, no significant differences were detected compared to the control pericytes, until late time points of co-culture with GBM cells (Figure 1B). Importantly, the increase in the production of TNF-α, which showed just a doubling of its expression at 72 hours of co-culture, was much lower than the one detected when assessing the anti-inflammatory cytokines TGF-β and IL-10 (Figure 1B). These data correlated with similar changes in the levels of mRNA expression in GBC-PC compared to controls (Figure 1C). Analysis of cytokine gene expression in GBC-PC reflected upregulation of the mRNA of the anti-inflammatory cytokine genes *Tgfb* and *Il10*. *Il1b and Il12* gene expression was not detected even in control pericytes (not shown), and the mRNA level of *Tnfa*, *Il4* and *Il23* in pericytes was not significantly affected by GBM cells. Surprisingly, mRNA and protein expression of the angiogenic cytokine IL-6 was clearly increased in GBC-PC compared to control pericytes after 72 hours of coculture (Figure 1C, Supplementary Figure 1) [30, 31]. No cytokine mRNA or protein were detected in control GBM cells (not shown). To determine if the marked rise of expression of TGF- β and IL-10 in GBC-PC requires direct cell-cell interaction or is mediated by soluble molecules expressed by GBM cells [32, 33], we incubated pericytes with sequential dilutions of supernatants from different lines of GBM cells. Our results showed the same levels of cytokine expression in supernatant-treated pericytes as in control pericytes, supporting that the acquired immunomodulatory phenotype in pericytes in response to GBM likely requires cell-to-cell interaction (Figure 1D).

### Pericytes express an immunosuppressive pattern of surface membrane molecules in response to interaction with GBM cells

**Figure 2:**
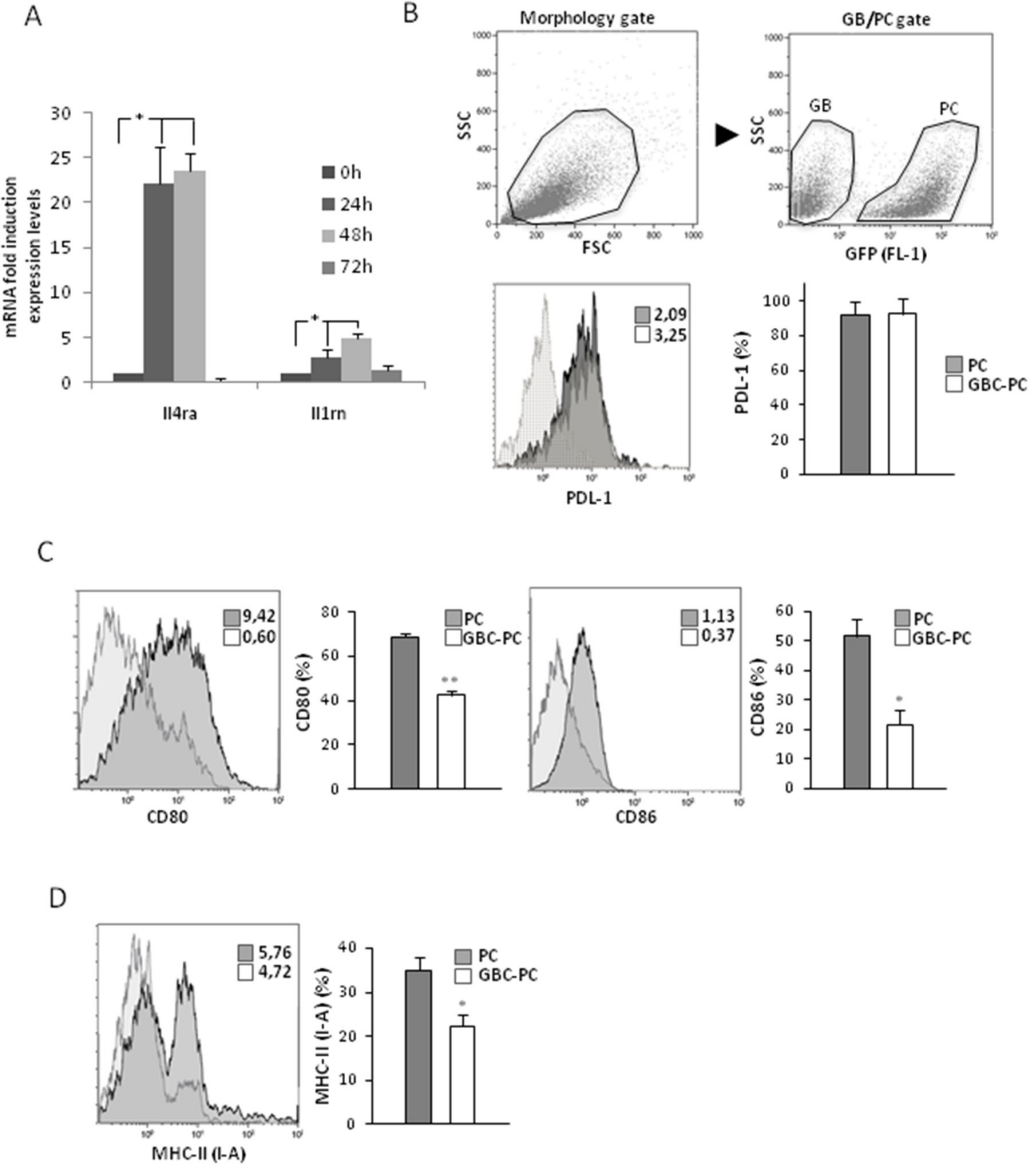
Pericytes express an immunosuppressive pattern of surface membrane molecules in response to interaction with GBM cells **(A)** Quantitative analysis of mRNA expression of Ilrn (IL-1RA) and Il4ra (IL-4RA) cytokine receptors in GBC-PC. Data are presented relative to basal levels in control pericytes at each time point of culture, and normalized to the housekeeping reference gene expression. Results are mean ± Standard Deviation obtained from at least, four independent experiments, * p<0.01. **(B)** Flow cytometry analysis in control PC or GBC-PC for PDL-1 expression after 72 h. Gating strategy for GFP^+^ pericyte population for further flow cytometry analyses is shown (upper panel). Solid and dotted histograms represent staining with PDL1 specific antibody and isotype control, respectively. Insert numbers inside histograms represent mean fluorescence intensity values. The bar graph represents percentages of expression of PDL-1 in PC compared to percentages in GBC-PC. **(C)** Flow cytometry analysis of expression of the co-stimulatory molecules CD80 and CD86. **(D)** Flow cytometry analysis of expression of MHC-II (I-A). Non-specific fluorescence was measured using specific isotype monoclonal antibodies and GBM cells were used as negative controls. All data of flow citometry represents mean ± Standard Deviation obtained from at least, three independent experiments, * p<0.05, or ** p<0.01.

Activated pericytes have been reported to present properties of myeloid cells, such as macrophages, expressing macrophage markers and acquiring phagocytic activity and the ability to present antigens to T cells [17, 18, 34]. To identify if pericytes might gain some of the immunosuppressive properties of TAMs in response to their interaction with GBM cells, we first analyzed the expression of several membrane molecules implicated in the inhibition of anti-tumor responses [24, 35]. Interestingly, we found high levels of *Il4ra* and *Il1rn* mRNA expression in pericytes, after 24 hours following interaction with GBM cells (Figure 2A). Then we determined if the immunosuppressive ligand of PD-1, PDL-1, which has been associated with glioblastoma progression [3, 36, 37], was expressed in pericytes, and if its levels changed in response to GBM interaction. We observed that PDL-1 was expressed in pericytes in resting conditions, but its level of expression was maintained upon GBM cell interaction (Figure 2B). Interestingly, we found that expression of the co-stimulatory molecules CD80 and CD86 was significantly reduced in GBC-PC compared to control pericytes (Figure 2C). To analyze if the ability of brain pericytes to present antigen to T cells might be affected by GBM cell interaction, we determined the expression levels of major histocompatibility complex class II molecules (MHC-II) in GBC-PC. We found a drastic and significant reduction of MHC-II expression in pericytes when cocultured with GBM cells compared to control pericytes (Figure 2D). None of these markers was detected in control GBM cells. These data support an immunomodulatory phenotype in pericytes in response to GBM cell interaction.

### GBM conditioned pericytes reduces T cell responses

**Figure 3:**
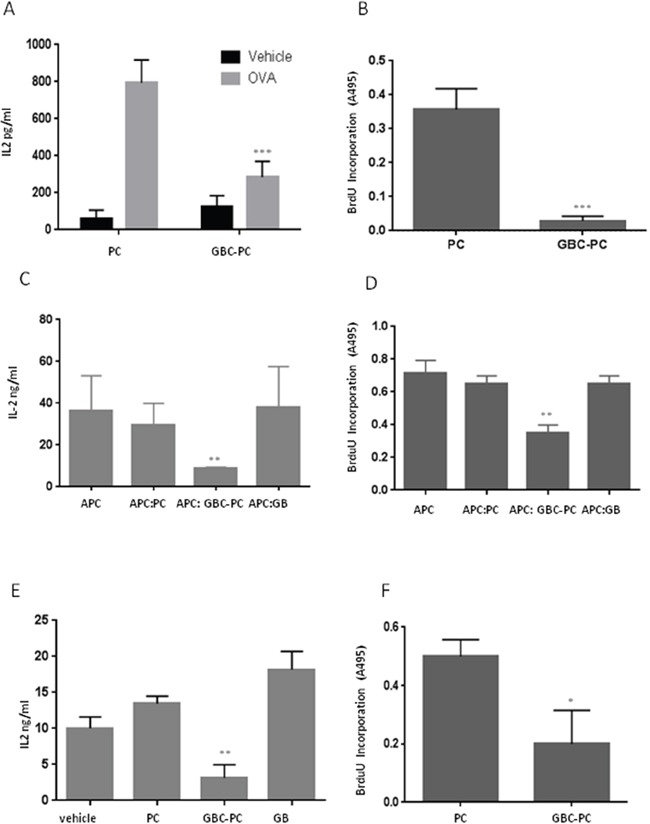
GBM conditioned pericytes reduce anti-tumor T cell responses **(A)** IL-2 production was measured by ELISA in resting and 72-hours stimulated naïve CD4^+^ T cells in response to OVA_323-339_ peptide presented by control pericytes (PC) and GBC-PC. Results are mean + Standard Deviation from five to six different experiments, ^***^p<0.005. **(B)** T cell proliferation was measured by BrdU incorporation in response to antigen presentation by PC and GBC-PC. Results are mean + Standard Deviation from four different experiments, ^***^p<0.005. **(C-D)** Cell proliferation and IL-2 production were measured in resting and 72-hours stimulated naïve CD4^+^ T cells in response to antigen presentation by antigen presenting cells (APC) in presence or not of GBC-PC (APC:GBC-PC; APC). T cells ractivaton in response to antigen presentation was also measured in the presence of control Pericytes (APC:PC) and GBM cells (APC:GB). Results are mean + Standard Deviation from four different experiments. **p<0.01. **(E-F)** Cell proliferation and IL-2 production were measured in naïve CD4^+^ T cells stimulated for 72 hours with plate-bound anti-CD3 (αCD3) and anti-CD28 (αCD28) antibodies, in the presence or absence of media from GBC-PC diluted 1:2 in fresh pericyte media (vehicle). Control media from pericytes (PC) and GBM (GB) cell cultures was also diluted in fresh pericyte media and cytokine production and cell proliferation measured in these control conditions. Results are mean + Standard Deviation from three different experiments. **p<0.01. *p<0.05.

It has been reported that pericytes activated by inflammatory challenge have the ability to present antigen on MHC molecules to T cells, regulating the activity of different T cells populations [16, 20, 21]. We observed that pericytes showed reduced expression of MHC-II molecules and an anti-inflammatory phenotype upon interaction with GBM cells. Therefore, we determined if, in response to GBM interaction, pericytes could instead impair T cell activation. For that purpose, we analyzed the ability of pericytes to present OVA_323-339_ peptide to T cells, and compared CD4^+^ T cell responses to peptide presented by GBC-PC with responses to antigen presentation by control pericytes (Figure 3A–3B). We found that pericytes were able to present OVA peptide and activate CD4^+^ T cells, which led to IL-2 production and cell proliferation. However, pericytes that were interacting with GBM cells showed a significantly impaired ability to activate T cells (Figure 3A–3B). As we had observed that GBC-PC produced high levels of anti-inflammatory cytokines, cytokines, expressed immunosuppresive molecules such as PDL-1 and showed reduced expression of co-stimulatory molecules, we analyzed whether GBM-PC would also affect T cell activation in response to antigen presented by professional antigen presenting cells (APCs). This would support that, when in contact with GBM, pericytes might not only show a reduced ability to cross-present tumor antigens but could also hinder the function of APCs. Corroborating our hypothesis, whereas CD4^+^ T cells produced high levels of IL-2 and proliferated in response to antigen presented by APCs, those T cells showed defective IL-2 production and proliferation when activated by antigen-loaded APCs in the presence of GBC-PC. T cell activation was not affected by control GBM cells or control pericytes (Figure 3C–3D). To further support that GBC-PC could release anti-inflammatory cytokines to the media that could explain their effect on APC-mediated activation of T cells, we determined whether GBC-PC-conditioned media would also be able to affect T cell activation. We then measured T cell responses to T Cell Receptor (TCR) engagement and co-stimulation, following activation with anti-CD3 and anti-CD28 antibodies, in the presence or absence of culture media from GBC-PC for 72 hours. Importantly, CD4^+^ T cells showed reduced IL-2 production and proliferation upon TCR and co-stimulation engagement in the presence of GBC-PC-conditioned media when compared to CD4^+^ T cells activated in presence of control pericyte media or control vehicle. IL-2 secretion and cell proliferation were not affected by control GBM cells (Figure 3E–3F). Importantly, impaired T cell responses caused by GBC-PC were not due to increased apoptosis, as there were no significant differences in cell death percentages between resting or activated T cells cultured with control pericytes or GBC-PC (Supplementary Figure 2). Therefore, our data support that GBC-PC reduces T cell responses through the induction of an anti-inflammatory response and the development of an immunosuppressive phenotype in response to interaction with GBM cells.

### Pericytes interacting with GBM cells assist tumor growth

**Figure 4:**
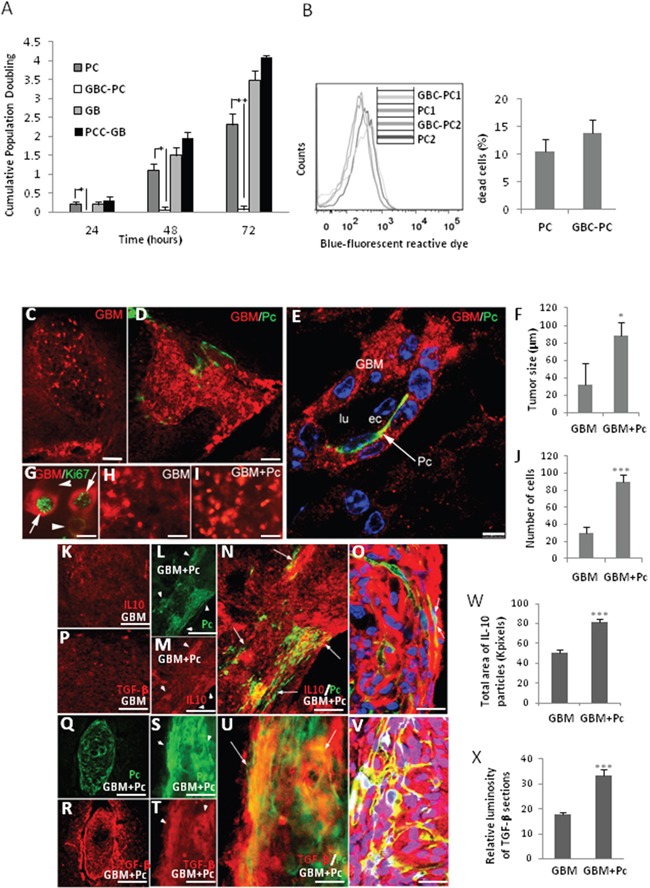
Pericytes interacting with GBM cells induce tumor growth **(A)** Proliferation of GBM cells interacting with pericytes (PCC-GB) and pericytes interacting with GBM cells (GBC-PC), represented as cumulative population doubling (CPD), during different times of co-culture was measured and compared to proliferation of control GBM cells and pericytes. Results are mean + Standard Deviation of at least three different experiments, *p<0.05; **p<0.01. **(B)** Detection of death cell by blue-fluorescent reactive dye, of two representative populations of pericytes (PC1, PC2) compared to those same populations conditioned by GBM cells (GBC-PC1, GBC-PC2) (left panel). Quantification of dead cells percentage (right panel) from at least, four independent experiments. **(C)** GBM tumor growth in mice that were xenografted with GBM cells (GBM) compared with **(D)** xenografts of co-cultured PC and GBM cells (GBM+Pc) (scale bars, 100 μm). **(E)** Infiltration stream of GBM cells (GBM) together with a GFP^+^ Pericyte (Pc) in the perivascular space (scale bar, 25 μm). **(F)** Morphometric measurement reveals the average of tumor size in GBM grafted mice (GB) and GBM+Pc grafted mice (6 tumors / 5 grafted mice and 7 tumors / 5 grafted mice, respectively). **(G)** Representative detail of xenografts GBM+Pc shows proliferating GBM cells (GBM/Ki67^+^), which appear as intense RFP^+^ fluorescent spherical dividing cells, in which one of the sister cells is more intensely immunopositive (arrow) than the other (arrowhead) for both RFP (red) and Ki67 (green) (scale bar, 25 μm). **(H)** Proliferating cells (strong RFP^+^) in GBM xenografts and **(I)** GBM+Pc xenografts (scale bars, 50 μm). All results are shown using the U373 GBM line, 4 weeks after-graft, and are representative of at least four different experiments using U87 or U373 GBM lines independently. Control pericytes (0 tumors / 5 grafted mice (4weeks) and 0 tumors/ 5 grafted mice (11weeks)) not shown. **(J)** Relative quantification of number of dividing GBM cells in GBM+Pc and GB grafted mice. Results are mean+SD from at least four different experiments * p<0.05; *** p<0.0005. **(K-P)** IL-10 and TGF-β expression in control GBM xenografts (scale bars, 50 μm). **(L, Q, S)** Colocalization of the heterogeneous distribution of pericytes (GFP^+^, arrowheads) and **(M, R, T)** IL-10 and TGF-β expression (arrowheads) in GBM+Pc xenografts. **(N, U)** Merged pictures showing higher IL-10/ TGF-β expression in areas where grafted GBC-PC accumulate (arrows) (scale bars, 50 μm). **(O, V)** High-power confocal image (one micron-thick section) shows the colocalization of GFP and IL-10/ TGF-β in GBC-PC (arrows) (scale bars, 50 μm). Results are shown 4 weeks after-graft and are representative of at least, four independent experiments. **(W)** Quantification of IL-10 expression relative to total area of IL-10 immune-positive particules in GBC+Pc xenografts compared to GB control. **(X)** Relative quantification of TGF-β expression related to luminosity of TGFb immuno-processed sections in GBC+Pc xenografts compared to GB control. Results are mean+SD from at least four different experiments * p<0.05; *** p<0.0005.

To determine if proliferation of GBM cells might be directly facilitated by pericytes, therefore enhancing tumor growth, we measured cell proliferation as cumulative population doublings in GBM cells interacting with pericytes *in vitro*. Interestingly, we found that GBC-PC did not proliferate at all, as opposed to control pericytes that proliferated, as expected, exponentially with the time of culture (Figure 4A). However, proliferation of GBM cells was not inhibited by the presence of pericytes (PCC-GB). PCC-GB seemed to reach even higher proliferation levels than control GBM cells, although this difference was not significant. In addition, we analyzed the cell survival of pericytes to confirm that the obtained data were due to inhibition of proliferation and not increased cell death. We found that pericytes survived after interaction with GBM cells during long time cultures (72 hours) as well as control pericytes (Figure 4B).

To confirm *in vivo* these findings, and corroborate that tumor growth might be assisted by GBC-PC with immunosuppressive properties, GBM cell proliferation *was* studied in grafts of co-cultured human RFP-GBM cells and GFP-mouse pericytes (GBM+Pc) grafted into brain cortex of an immunocompetent C57Bl/6 mouse model [25]. GBM tumors from both U373 and U87 cell lines (clonally labelled by RFP expression), were observed in most of the grafts with GBM cells alone (control grafts) or GBM+Pc at 4 and 11 weeks after graft (Figure 4C, 4D). Interestingly, pericytes were not observed when pericytes were grafted alone or together with GBM cells but without prior co-cultured (control grafts). As expected, these control mice showed the same tumor progression than mice grafted with glioblastoma alone (not shown). Increased perivascular infiltration of GBM cells was observed in GBM+Pc grafts (Figure 4E). The GBM tumor mass was larger in the brain of mice xenografted with GBM+Pc than in mice xenografted with GBM cells alone (Figure 4C, 4F). Due to the ovoid-like shape of the tumor mass we measured the tumor diameters in the central section and calculated the volume of the tumoral ovoid: 5.8+/−0.5×10^6^ μm^3^ in GBM+Pc grafts [n=5] and 2.5+/−0.3×10^6^ μm^3^ in GBM grafts [n=4] (Figure 4F). Supporting this data, we observed that dividing GBM cells, which appear as spherical strong RFP^+^ Ki67^+^ cells (Figure 4G), were more abundant in GBM+Pc grafts than in GBM grafts (Figure 4H, 4I). Relative quantification (counting strong fluorescent RFP cells in the same area of two consecutive sections of GB+PC grafts [90+/−10; n=5] and GBM grafts [30+/−7; n=5]; ImageJ-NHI software) showed that dividing cells were three times more numerous in GBM+Pc than in GBM grafts (Figure 4J). To determine if xenografts of GBM+Pc were able to acquire an anti-inflammatory phenotype *in vivo*, we analyzed the expression of IL-10 and TGF-β on the tumor/brain edge. Compared to GBM cell grafts (Figure 4K, 4P), we detected an increase of IL-10 and TGF-β expression in GBM+Pc grafts (Figure 4L–4M and 4Q–4TQ, respectively). This expression was particularly high in areas where grafted GBC-PC were identified surrounding blood vessel. The most intense fluorescent areas of Cy5-IL10 and Cy5-TGF-β colocalized with GFP expression corresponding to PC cellular mass (Figure 4N–4O and 4U–4VU, respectively). Quantitative analysis revealed that the expression of IL-10 (by measuring the area of immunopositive particles in equivalent areas of the central section of each xenograft [n=5] using ImageJ-NIH software) and TGF-β (by quatifying luminosity in the same area of the central section of each xenograft [n=5] using Adobe Photoshop software) were significantly higher in GBM+PC grafts than in GBM control grafts (Figure 4W and 4X, respectively). Supporting these data, we detected the appearance of inhibitory PD-1/PDL-1 interactions, which corresponded to perivascular lymphocytic infiltration and pericytes respectively in GBM+Pc grafts (Supplementary Figure 3), and compared it to host pericytes, where we did not find those interactions though perivascular PDL- 1 expression was present (not shown). Interaction between PD-1 receptor in lymphocytes and PDL-1 in GBM progression has been described, but it is not clear if, apart from GBM cells, other cell types may also present PDL-1 in perivascular areas [36]. Our data showed pericytes expressing PDL-1 at the plasma membrane and interacting with PD-1 expressed in perivascular infiltrating T cells. Unspecific detection of high membrane-associated expression of PDL-1 was also detected in perivascular areas that did not correspond to pericytes but to GBM cells [36, 37] (Supplementary Figure 3).

## DISCUSSION

Cellular interactions in the tumor microenvironment have been associated to immunosuppression and the induction of tolerance in the immune system against the tumor. In the immunosuppressive niche, in addition to cancer cells, tumor-associated macrophages (TAMs), glia cells, endothelial cells and T cells also infiltrate the tumor and secrete cytokines, chemokines, and proteases, thus promoting tumor angiogenesis, growth, metastasis, and immunosuppression [24]. The inability to clear the tumor is known, in part, to be a consequence of an immunosuppressive response, with high levels of anti-inflammatory cytokines and reduced expression of T cell activation molecules [35, 38]. However, the mechanisms underlying that immunosuppressive response are not well understood yet, and even less in perivascular areas where tumor cells can infiltrate and metastasize. In this work, we describe a previously unknown immunosuppressive role of brain perivascular pericytes and demonstrate the importance of pericytes’ interaction with tumor cells during GBM progression. Our study reveal that brain pericytes show a pattern of immunosuppressive membrane surface molecule expression in response to GBM cell interaction. Analysis of cytokine expression from GBC-PC also shows high levels of expression of the anti-inflammatory cytokines IL-10 and TGF-β, low level of the pro-inflammatory cytokine TNFα and hardly any production of other important pro-inflammatory cytokines compared to basal levels detected in control pericytes. Interestingly, the increased production of anti-inflammatory cytokines correlates with an upregulation of those cytokines’ gene expression in pericytes, suggesting that pericytes are conditioned by GBM cells (GBC-PC) to produce those cytokines. Our findings indicate that *Il1b* and *Il12* gene expression was not even detected in control pericytes (not shown), and that the mRNA level of *Il4, Tnfa* and *Il23* in pericytes was barely affected by GBM cells. Although protein levels of TNFα are increased in GBC-PC compared to control pericytes, the doubling in amount of protein expression of this pro-inflammatory cytokine (reaching up to 45 pg/ml approximately) is much lower than the protein expression of the anti-inflammatory cytokines TGF-β and IL-10, which reaches higher levels of up to 100 and 400 pg/ml, respectivately in GBC-PC. Interestingly, mRNA and protein expression of the angiogenic cytokine IL-6 released by pericytes in response to neurovascular damage [30, 31] was surprisingly raised in GBC-PC. In a recent study in ovarian cancer, IL-6 was shown to induce defective angiogenesis through altered pericyte coverage in aortic ring vessels [31]. Interestingly, treatment of pericytes with TGF-β has been recently associated with regulation of the neurovascular function, affecting the phagocitic ability of these cells, reducing pericytes proliferation and increasing IL-6 expression [19]. The fact that IL-6 has been associated to an angiogenic gene program in pericytes and even affects pericytes proliferation during angiogenesis in cancer, supports our data and might also explain why we observed a reduced proliferation of GBC-PC *in vitro*. It is possible that IL-6 expression might be regulated by TGF-β signaling in GBC-PC, attenuating cell proliferation and other neurovascular functions that also affect the tumor progression.

It has been recently shown that GBM cells might interact with host cells, such as pericytes, to transfer malignant properties and affect their function [8, 9], supporting that GBM-pericytes cell-cell direct interaction are required to lead the changes in the pericytes immune phenotype.

Our hypothesis that GBC-PC might present similar immunosuppressive properties as TAMs, which are implicated in inhibitory anti-tumor responses, is supported by the clear upregulation of *Il4ra* and *Il1rn* gene expression in GBC-PC [24, 35]. Indeed, our results show that PDL-1, which is associated with glioblastoma progression and contribute to suppress anti-tumor T cell responses [3, 36, 37, 39], is expressed in pericytes and maintained upon GBM interaction. Furthermore, we show reduced expression of T cell co-stimulatory molecules, such as CD80 and CD86, in GBC-PC, supporting an immunosuppressive phenotype that might prevent tumor clearance. Our data confirm that brain pericytes express MHC molecules [16, 20, 21], but interestingly expression levels are drastically reduced in GBC-PC compared to PC, suggesting that the anti-tumor T cell response might be affected through inefficient antigen presentation. GBC-PC also shows impaired ability to present antigen to T cells and cell culture media from GBC-PC is enough to reduce T cell responses. In addition, T cell function in response to antigen presentation by APCs is also suppressed in the presence of GBC-PC. Therefore, these results suggest that GBC-PC could acquire suppressing properties and hinder T cell activation, contributing to tumor growth by preventing the activity of other APCs.

Brain pericytes preferentially cover ECs junctions in response to inflammation, having an important role in blood brain barrier disruption in inflammatory processes [28, 40]. Interestingly our studies reveal that GBC-PC remain in a dormant-like state and do not proliferate compared to control pericytes, which proliferate normally. In contrast, proliferation of GBM cells interacting with pericytes (PCC-GB) is not affected.

Supporting our *in vitro* studies, we demonstrate *in vivo* that GBM cell proliferation and, therefore, tumor progression, is assisted by the interaction with pericytes. Our results strongly suggest that GBM cell interaction with pericytes is required to maintain immunotolerance in the immunocompetent mouse brain [25]. Indeed, our results analysing GBM+Pc grafts compared to control grafts, show high levels of anti-inflammatory cytokines in perivascular areas where grafted GBC-PC were found. Perivascular detection of less intense anti-inflammatory cytokine levels in GBM grafts might correspond to expression by endogenous pericytes.

Supporting our *in vitro* results, we have also seen the establishment of inhibitory interactions PD-1/PDL-1 between lymphocytes and pericytes, respectively, in perivascular areas. Interestingly, we don’t detect either the presence of grafted pericytes in grafts of control mice, or when pericytes are grafted with glioblastoma cells without being previously co-cultured. As it was expected, these last control mice showed the same tumor progression that control mice grafted with glioblastoma alone. However, we observe pericytes in grafted GBM+Pc mice, indicating they need to be conditioned by interaction with Glioblastoma previously to anchor in perivascular areas. This effect may be due to a decreased competence of grafted versus host pericytes for trophic factors to survive; and suggests that GBM cells confer increasing competence to grafted pericytes, as well as inducing immunotolerance, which in addition could facilitate the cell anchorage to perivascular areas.

In conclusion, our results indicate that brain pericytes show an immunosuppressive function as a consequence of their interaction with GBM cells, possibly assisting the tumor immune evasion and, consequently promoting GBM tumor. This finding identify pericytes as key cellular components of the GBM niche, which should lead to further studies to search for possible ways to regulate their interaction with GBM cells to modulate their immunosuppressive function and control tumor growth.

## MATERIALS AND METHODS

### Mice

Six to eight-week-old wild type C57Black/6, C57Bl/6-Tg(ACTB-EGFP)1Osb/J (Charles River laboratory) and OT II TCR transgenic (Jackson laboratory) mice were maintained in pathogen-free conditions in the animal facility of University of Murcia. All animal procedures were approved and performed according to the guidelines set by University of Murcia Institutional Animal Care and Use Committee.

### Cell culture

Primary brain pericytes from GFP-actin mice were isolated as described previously and according to the method of Oishi et al. [9, 26, 27]. Pericytes were used from 5^th^-9^th^ passage [27] and checked by pericytes markers. For the detailed cell culture, please see Supplementary Text.

Human glioblastoma cell lines U373-MG and U87 were purchased from European Collection for Authenticated Cell Cultures (ECACC). Both lines expressing RFP protein were used for cell tracking. For the detailed cell cloning, please see Supplementary Text.

Co-cultures of pericytes and GBM cells, at a ratio of 1:1, were plated in pericytes media for 24-72 hours. Cells were trypsinized, replated and identified by GFP- or RFP-protein labeling. Pericytes were sorted by GFP-protein with a Cell Sorter (Sony SH800).

Primary CD4^+^ T cells were isolated from lymph nodes and spleens of mice using anti-CD4-coupled magnetic beads (Life Technologies). In some cases, isolated T cells were stimulated with 0.5 μg/ml plate bound anti-CD3 and 0.5 μg/ml anti-CD28 (BD Biosciences). CD4^+^ T cells were growth in T cell media.

### Real-time PCR (qPCR)

cDNA was synthesized from total mRNA, and gene expression was analyzed by real time PCR using SYBR Green in a Step One Plus Thermocycler (Applied Biosystems). Gene expression was normalized to mouse β-actin expression. Glioblastoma cells were used as negative control for cytokines expression. For primer sequence information, please see Supplementary Text.

### Functional assay of pericytes

Co-cultures of pericytes (1 × 10^5^) and GBM cells in 1:1 proportion, were plated in 96 well plate. Pericytes and GBM cells cultures were used as controls. After 72 hours, T cells (5×10^4^) were plated on attached pericytes or attached GBC-PC previously, in 96 well plate, in 5:1 proportion respectively and in presence or not of OVA_323-339_ peptide antigen (Sigma-Aldrich). Pericytes were detached with trypsin 0.05% after 72 hours and plated with T cells at the same time to confirm the same results. For suppression assay of T cell function, splenocytes and T cells were plated in 96 well plates, in 5:1 proportion, in presence or not of OVA_323-339_ peptide on GBC-PC or pericytes plated 72 hours before. In some cases, media from cultures of GBC-PC, control pericytes and control GBM cells for 72 hours, was recollected to add to stimulated T cells with plate-bound antibodies.

### ELISA

Pericytes (5×10^4^) were cultured with GBM cells at 1:1 proportion in 96-well plates for 24-72 hours. Supernatants were collected, and TGF-β, IL-10, TNF-α and IL-6 levels were measured by sandwich ELISA with specific anti-mouse antibodies following manufacturer’s recommendations (Diaclone and Elabscience, respectively). IL-2 levels from T cell supernatants were measured in a sandwich ELISA following manufacturer’s recommendations (BD Biosciences).

### Flow cytometry analysis

Expression of PDL-1 (R&D Systems), CD86, CD80 (eBioscence) and I-A [2] (MHC/H2 class II histocompatibility molecules; BD Bioscience) were analyzed using specific anti-mouse antibodies. Non-specific fluorescence was measured using specific isotype monoclonal antibodies and GBM cells as negative control. Pericytes death was determined using (LIVE/DEAD Fixable Blue Dead Cell Stain Kit, ThermoFisher). Stained cells were analyzed by flow cytometry using a FACS Canto II Flow cytometer (BD Bioscience) and data were analyzed with Kaluza analysis software (Beckman Coulter, Fullerton, CA).

### Proliferation assay

Pericytes (5×10^4^) were cultured with GBM cells at 1:1 proportion in 6-well plates for 24-72 hours. After, pericytes and GBM cells were isolated by GFP or RFP sorting, respectively or only trypsinized to break cell interactions. GBM cells interacting with pericytes were defined as PCC-GB and pericytes interacting with GBM cells as GBC-PC. Both types of cells were counted using an inverted fluorescence microscope (Nikon, Tokyo, Japan). Cumulative population doubling level was calculated using the formula: “$\text{PD}=\left(\frac{1}{\mathrm{Log}102}\right)x\mathrm{Log}10(\frac{Nt}{No})$” where No is the number of viable cells (as determined by trypan blue exclusion) at seeding, whereas Nt is the number of viable cells at harvest.

T cells stimulated for 72 hours in functional pericytes assays were transferred to 96 well plate in order to separate from adherent GBC-PC or control pericytes and GBM cells. BrdU was added for 12 hours. After, incorporation of BrdU was measured by ELISA according to the manufacturer’s instructions (Roche).

### Xenografts

Cell pellets from human GBM cells and/or murine pericytes that were co-cultured for 72 hours (5×10^6^ cells), were grafted into C57Black/6 wild type mice brains. Xenografts (10 mice received GBM, 10 mice received GBM+PC, 10 mice received PC and 10 mice received GBM/PC without being co-cultured previously) were performed as described previously [9] in an immunocompetent mouse model [25]. Cell pellets, prepared as hanging drops, were grafted into mice. Xenografts (1 pellet/mouse) were introduced into the right hemisphere through a small craniotomy (2 to 3 mm from the midline, approximately 1 mm behind the bregma) at 2.5 mm depth, using a stereotactic apparatus and a Pasteur pipette hand-pulled to an internal diameter of 0.38 mm. This produced grafts that integrated into the cortex or the hippocampus. After 4 and 11 weeks post-grafting, mice were perfused using 4% paraformaldehyde (5 mice of each experimental grafts at each fixation time). Brains were embedded in 30% sucrose and cut at 40 μm using a cryostat. For Immunohistochemistry methods and the antibody information, please see Supplementary Text.

### Statistical analysis

Differences between groups were analyzed by one way ANOVA with a Tukey-kramer post-test. Comparisons between data pairs were analyzed using a *t* test. Statistical significance was defined as p<0.05.

## SUPPLEMENTARY MATERIALS FIGURES


