# Supplementary material for: Glioblastoma progression is assisted by induction of immunosuppressive function of pericytes through interaction with tumor cells
Source: Oncotarget. 2017 Aug 2;8(40):68614–26. doi: 10.18632/oncotarget.19804 (PMC5620282; doi:10.18632/oncotarget.19804)
Supplement: Supplementary file 1 [file oncotarget-08-68614-s001.pdf]

Glioblastoma progression is assisted by induction of immunosuppressive function of pericytes through interaction with tumor cells

SUPPLEMENTARY MATERIALS

Cell culture

For co-culture experiments, primary brain pericytes from GFP-actin mice were isolated as described previously and according to the method of Oishi et al. (22). This protocol is based in the isolation of perivascular stem cells (pericytes) from brain venules and arterioles. Pericytes were expanded and used from 5th passage to 9th passage to make sure the elimination of any possible contamination of endothelial cells in a cell culture media without specific growth factors (22,23). Pericytes were growth in α-MEM medium supplemented with nucleosides, 20% fetal bovine-serum (Gibco Invitrogen, Paisley, Scotland) and 2 mM L-glutamine. In any case, pericytes from 5th-9th passage were trypsinized, replated and checked for double-labeling using pericytes markers. Pericytes culture purity was confirmed by staining with the following specific anti-mouse antibodies: rabbit-NG2 chondroitin sulphate proteoglycan (Chemicon), goat-RGS5 (Santa Cruz), rabbit-PDFGRβ (Bioss), mouse-alpha Smooth Muscle Actin (αSMA; Abcam). Absence of astrocytes, endothelial or microglia cells in the pericytes culture was checked with the following antibodies, respectively: rabbit-glial fibrillary acidic protein (GFAP; Chemicon), hamster-CD31 (Abcam), rat-CD11b (Mac-1; BD Pharmingen).

Glioblastoma cells (GBM) were grown in α-MEM medium supplemented with 10% fetal bovine serum. For cell tracking, cells were transfected with the cDNA fragment encoding the leader sequence and GPI signal of the human decay-accelerating factor (CD55 molecule, NM_000547), that was fused in frame with the coding sequence of the red fluorescent protein (RFP). The fusion protein was subcloned into BamHI and NotI digested pIRES1neo3 vector.

Co-culture experiments with GBM and pericytes were carried in media to growth pericytes. Supernatants from different GBM cell lines (U373, U87) that were plated in fresh pericytes media in the same concentration as co-culture of GBM and pericytes, were recollected and spin to eliminate rest of cells (500xg), after 72 hours. Total supernatants and diluted supernatants in fresh pericytes media (to avoid changes of FBS or absence of any nutrient) were added to pericytes during 72 hours more.

$CD4^+$ T cells were maintained in exponential growth in DMEM supplemented with 10% FBS, 2 mM L-glutamine, non-essential amino acids (Cambrex), essential vitamins (Cambrex) and 50 μM 2-mercaptoethanol. Apoptosis of T cells was determined with an Annexin V-PE apoptosis detection kit (BD Biosciences). Stained cells were acquired by flow cytometry.

qPCR

to assess levels of cytokines expression, the following primers (Sigma Aldrich, St. Louis, MO) were used: mouse *Tgfb* 5′-CTCCCGTGGCTTCTAGTGC-3′ and 5′-GCCTTAGTTTGGACAGGATCTG-3′; mouse *Il10*, 5′-GCTCTTACTGACTGGCATGAG-3′ and 5′-CGCAGCTCTAGGAGCATGTG-3′; mouse *Tnfa* 5′-CCCTCACACTCAGATCATCTTCT-3′ and 5′-GCTACGACGTGGGCTACAG-3′; mouse *Il6,* 5’-TAGTCCTTCCTACCCCAATTTCC -3’ and 5’-TTGGTCCTTAGCCACTCCTTC -3’; mouse *Il23*, 5′-ATGCTGGATTGCAGAGCAGTA-3′ and 5′-ACGGGGCACATTATTTTTAGTCT-3′; mouse *Il12*, 5′-CTGTGCCTTGGTAGCATCTATG-3′ and 5′-GCAGAGTCTCGCCATTATGATTC-3′; mouse *Il1b* 5′-GCAACTGTTCCTGAACTCAACT-3′ and 5′-ATCTTTTGGGGTCCGTCAACT-3′; mouse *Ilrn*, 5′-GCTCATTGCTGGGTACTTACAA-3′ and 5′-CCAGACTTGGCACAAGACAGG-3′; mouse *Il4ra*, 5′-TCTGCATCCCGTTGTTTTGC-3′ and 5′-GCACCTGTGCATCCTGAATG-3′. Mouse (5’-AAGGACTCCTATGTGGGTGACGA-3’; 5’-ATCTTCTCCATGTCGTCCCAGTTG-3’) and human (5’-GTCTGCCTTGGTAGTGGATAATG-3’; 5’-TCGAGGACGCCCTATCATGG-3’) β-Actin expression was measured as reference housekeeping controls.

Xenografts immunohistochemistry and microscopy

In cryostat sections we used the following antibodies: rabbit-RFP (MBL), rat-RFP (Chromoteck), chicken-GPP (AVES), rabbit-Ki67 (Thermo Scientific), rabbit anti-mouse IL-10 (Bioss), mouse-TGF-β1,2,3 (R&D systems), goat anti-mouse PDL-1 (R&D Systems), rabbit-PD-1(Novus). Single and double labelling patterns were revealed by fluorescence microscopy, using corresponding secondary antibodies (supplementary text). coupled to Alexa488, Alexa594

or Cy5 (Invitrogen/Molecular Probes, respectively), and biotinylated secondary antibodies (VectorLabs), followed by Cy3-streptavidin reagent (Amershan-GEHealthcare). All fluorescence samples were counterstained with DAPI (Invitrogen/Molecular Probes) prior to mounting with Mowiol. A TCS-SP2-AOBS laser scanning spectral inverted Confocal Microscope (Leica Microsystems, Barcelona, Spain) was used for analysis of histological sections. A fluorescence automated DM6000B microscope and a MZ16FA Fluorescence Stereomicroscope (for wide-field microscopy), running Leica Application Suite (LAS) AF6000 Software (version 2.0.2), equipped with a DFC350-FX (monochrome) or DC500 (color) digital cameras (all purchased from Leica Microsystems, Barcelona, Spain) were used to analyze fixed cells and histological sections from xenografts. Morphometric measurements and quantification of cells by fluorescent imaging were performed using Image J-NIH, USA software and Adobe Photoshop. Pictures for illustrations and quantitative analysis were uploaded from direct microscopic images and were not manipulated in subsequent steps of figures preparation, except for framing and scaling.

Intensity of IL10 immunoreaction was measured using particle analyzer in ImageJ program. We have uploaded into the program the sections that have been used for volumetric calculation for each graft, and, standardizing all the image settings in GBM and GBM+Pc sections, and analyzing total area of immuno-positive particles. Since TGF-β immunoreactivity particles in GBM grafts were very infrequent in the sections, we have decided to analyze luminosity of the red channel by using Photoshop program (inside Histogram information), analyzing the same number of pixels (around 3500 pixels) in standardized uploaded images from GBM and GBM+Pc sections.

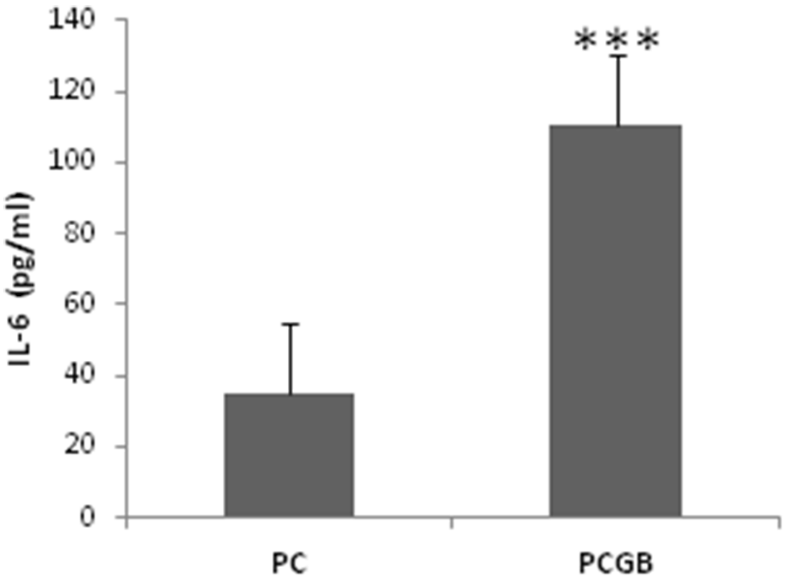

Supplementary Figure 1: ELISA of IL-6 cytokine levels in pericytes co-cultured with Glioblastoma cells (GBC-PC) at 72 hours and compared to cytokines basal levels in control pericytes (PC). *** $p<0.001$. All data represents mean ± Standard Deviation obtained from at least, four independent experiments.

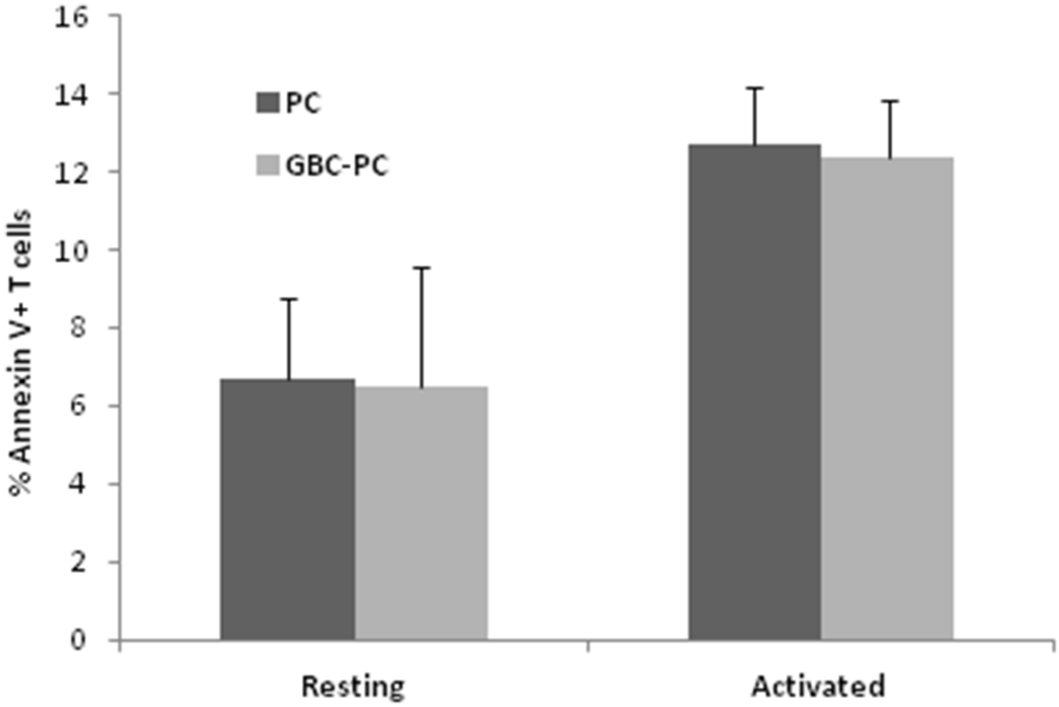

Supplementary Figure 2: Quantification of Annexin V⁺ cells in resting and activated CD4⁺ T cells in response to antigen presentation by pericytes (PC) and GBC-pericytes (GBC-PC).

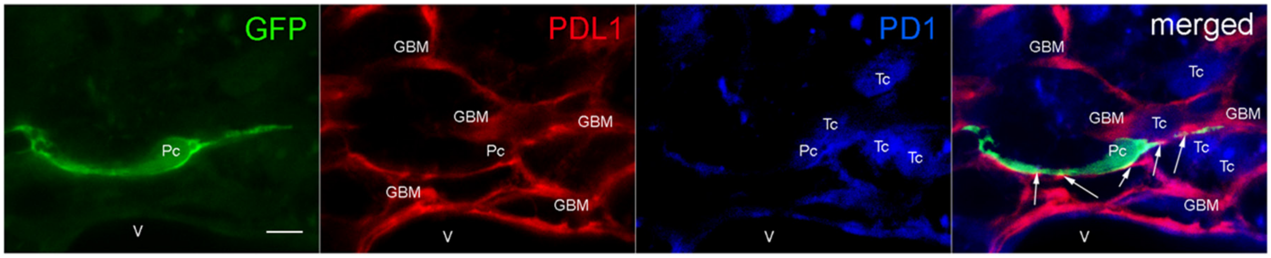

Supplementary Figure 3: Expression of PDL-1 ligand in perivascular pericytes (Pc) interacting with the PD1 receptor of lymphocytes (Tc) in Glioblastoma Multiforme (GBM) xenograft. Pericytes in green (GFP), PDL1 in red (far red), GBM-RFP cells (not shown), PD1 in blue. Arrows in the merged picture indicate the expression of PDL1 in pericytes membrane (colocalization appears in yellow color). Blood vessel (V). Scale bar 25 micrometres.
